# Impact of Forest Management on Species Richness: Global Meta-Analysis and Economic Trade-Offs

**DOI:** 10.1038/srep23954

**Published:** 2016-04-04

**Authors:** Abhishek Chaudhary, Zuzana Burivalova, Lian Pin Koh, Stefanie Hellweg

**Affiliations:** 1 Institute of Environmental Engineering, ETH Zurich, 8093 Zurich, Switzerland; 2 Woodrow Wilson School of Public and International Affairs, Princeton University, 08540 Princeton NJ, USA; 3 Environment Institute, and School of Biological Sciences, University of Adelaide, 5005 Adelaide, Australia

## Abstract

Forests managed for timber have an important role to play in conserving global biodiversity. We evaluated the most common timber production systems worldwide in terms of their impact on local species richness by conducting a categorical meta-analysis. We reviewed 287 published studies containing 1008 comparisons of species richness in managed and unmanaged forests and derived management, taxon, and continent specific effect sizes. We show that in terms of local species richness loss, forest management types can be ranked, from best to worse, as follows: selection and retention systems, reduced impact logging, conventional selective logging, clear-cutting, agroforestry, timber plantations, fuelwood plantations. Next, we calculated the economic profitability in terms of the net present value of timber harvesting from 10 hypothetical wood-producing Forest Management Units (FMU) from around the globe. The ranking of management types is altered when the species loss per unit profit generated from the FMU is considered. This is due to differences in yield, timber species prices, rotation cycle length and production costs. We thus conclude that it would be erroneous to dismiss or prioritize timber production regimes, based solely on their ranking of alpha diversity impacts.

Each year, wood valued over US$100 billion is removed from forests globally, mainly accounted for by industrial roundwood, and also including fuelwood^1^. Around 10 million people are employed in the forestry sector and the livelihoods of many more depend on forests^1^. At the same time, it is now clear that forests managed for timber have to play an important role in conserving global biodiversity, as strictly protected areas, where all extractive activities are prohibited, are simply insufficient to achieve global biodiversity conservation goals^2^,^3^. Preserving forest biodiversity without harming economic interests is a big challenge for nations with forests. Local biodiversity loss due to timber extraction activities can disrupt the long-term resilience of forests, which may in turn cascade into an impoverished delivery of ecosystems services, ultimately affecting also human well-being^4^,^5^.

With increasingly globalized trade, there is a growing choice of timber products from various tropical and temperate tree species^6^. Apart from aesthetic, financial and functional preferences, a small but growing group of customers is also increasingly aware of, and interested in the environmental impact associated with wood products from around the globe^7^. The increasing globalization of timber trade also means that the environmental impacts often occur far from the place of consumption^8^. Informing consumers about the biodiversity impacts “hidden” in the wood products could be an important step toward achieving sustainable consumption patterns.

The most commonly mentioned impacts of the timber industry is forest degradation^9^,^10^. Extracting timber or other products changes the tree age structure, composition of tree species and vertical stratification, thereby affecting local temperature, light, moisture, soil, and litter conditions. This results in changes or complete removal of microhabitats (such as dead wood, cavities, root plates or mature trees) that host forest biodiversity^11^. The magnitude of impact on species diversity depends on several factors. First, forestry is carried out in different climatic zones with different native biodiversity, food web structures, and ecosystem properties. Therefore forestry impacts have a large geographical variability. Second, within the same region, different taxonomic groups may respond in different ways to forestry operations due to variation in, for example, body size, mobility, and diet^12^,^13^. Third, some types of management may have a larger influence on forest species than others, due to differences in habitat structure and continuity, or microclimatic conditions after the harvest. Additionally, some management regimes might have stronger secondary impacts on biodiversity, such as through increased rates of hunting or fire occurrence.

At an extreme end of the management intensity range is clear-cutting, which results in temporary deforestation of a previously forested area. Reduced Impact Logging (RIL) is an example of harvesting technique at the other end of the timber production management spectrum. Under RIL, carefully selected individual trees are extracted from the forest by trained workers, according to a harvest plan and guidelines designed to minimize the deleterious impact of tree felling, hauling and yarding on the remaining forest^14^. A quantitative understanding of the impacts of different forest management regimes on biodiversity in different regions of the world is crucial to any efforts to reconcile biodiversity conservation and economic interests.

## Management types

We chose the most common timber producing forest management regimes to assess the response of species richness to them. Some management types that we considered could be further divided into finer categories and others merged into broader ones, or considered as techniques within another management category. We have striven to achieve a good balance between data availability and information loss from excessive generalization.

### Clear-cut (temperate and boreal)

Clear-cutting is historically the most common example of even-aged silviculture practice in temperate and boreal biomes^15^. It is technically easy to execute, as the entire stand overstorey is removed in one harvest. Clear-cutting has been criticized for simplifying forest structure and reducing biological diversity, leading to homogeneous forests^16^ (but see Greenberg *et al*. for exceptions, where clear-cutting is found to mimic high intensity natural disturbance regimes)^17^. Many countries are now abandoning this practice. The reduction of clear-cut areas is also a part of regulations and standards under many forest certification schemes^18^. We only consider clear-cutting of natural managed forests, rather than of plantations, and without any additional interventions.

### Retention (temperate and boreal)

In recent decades, silvicultural practices that combine timber harvesting and biodiversity preservation have been promoted to mitigate the impacts of clear-cuts^19^. This has led to other variations of even-aged silviculture, in which individuals (dispersed retention) or groups of trees (aggregated retention) are left on-site to maintain structural diversity (such as patch-cut or green tree retention systems), supply seeds for the next crop (seed tree retention) or to protect the regenerating understorey (shelterwood system)^18^,^19^. Ideally, this category will be subdivided in the future according to the amount and configuration of trees retained, to assess the impact of individual retention practices.

### Selection systems (temperate and boreal)

Selection system is a silvicultural program aimed to maintain uneven-aged stands and is applied as an alternative to clear-cutting^20^. It is designed to remove individual mature trees (single-tree selection), groups of mature trees (group-selection), or a combination of the two to create small openings scattered throughout the stand. This results in heterogeneous stand structures, which are assumed to be less damaging to forest biodiversity than traditional clear-cuts^20^. Selection systems place unique emphasis on maintaining species and structural diversity and regard such non-commodity values as a necessary foundation to achieve sustainable provision of timber and other ecosystem services^21^.

### Conventional selective logging (tropical)

Selective logging involves the removal of the largest, highest quality trees from a tropical forest stand, leaving the remaining vegetation standing^22^. The term selective logging encompasses a very broad range of interventions, varying in, for example, the intensity of extraction (ranging from <5 m^3^ha^−1^ to almost 200 m^3^ha^−1^), the use of bulldozers or cables to extract timber, legality, and ground disturbance^23^. On the one hand, high intensity selective logging can disturb as much as 30–40% of the area (through extensive skid trails and log landings), and damage 40–70% of remaining unlogged trees^24^. On the other hand, in areas with low density of commercially valuable trees, such operations can have a relatively low impact on the remaining forest stand^25^.

Note that we distinguish *selection systems* in temperate and boreal regions from *selective logging*, primarily practiced in the tropics, because we argue that selection systems are effectively an attempt towards sustainable forest management, whereas selective logging is typically the default option of timber harvest in the species-diverse tropics, as only certain timber species are of commercial interest.

### Reduced impact selective logging (tropical)

Reduced Impact Logging (RIL) techniques have been proposed in an attempt to manage tropical forests for timber in a more sustainable way by reducing the damage to soil, future crop trees, residual stands, and workers^14^. RIL includes preparation of detailed harvest plans, worker education, training and supervision, demarcation of log extraction paths, directional felling to reduce collateral damage to surrounding trees and canopy, and protection of riparian areas^14^. RIL has been conceived to achieve a sustainable level of harvest, but also to improve sustainability in terms of biodiversity, carbon retention and ecosystem services^9^. Even though RIL techniques can be practiced anywhere and within other management regimes including plantations, here we only consider studies reporting species richness in natural tropical forests managed using reduced impact selective logging.

### Timber plantations

Timber plantations are becoming increasingly popular in some regions as an alternative to extracting timber from natural forests^26^. Whereas some timber species are relatively easily grown in plantations, others have a very low survival rate in plantations (e.g. mahogany, rosewood). There are likely important differences, in terms of biodiversity, between monoculture and mixed timber plantations, plantations of native *vs*. exotic species, and in the way timber is harvested (clear-cutting, selective logging). However, there is currently insufficient number of studies to distinguish between these effects, and little is known about the dynamics of tropical timber growth in plantations.

### Plantations for fuelwood and pulp

A large proportion of plantations are monocultures of fast growing, low-density wood species, such as *Pinus spp*. or *Eucalyptus spp*., used for fuel, or the pulp and paper industry, notably *Acacia mangium*. These plantations also often consist of exotic species, and we consider them as a separate category, because they are typically harvested on a much shorter time cycle than timber plantations. We include this and the following non-timber producing management categories to provide a comparison with the above timber producing management types.

### Non-timber plantations

Industrial clearing of tropical forests for non-timber plantations (most importantly palm oil but also plantain, bamboo, or rubber, which is planted also for timber) has been a key driver of biodiversity loss in tropical countries^27^. For example, at least 55 % of the expansion of oil palm between 1990 and 2005 in Malaysia and Indonesia occurred at the expense of rainforest^28^. Monoculture rubber plantations are now the most rapidly expanding tree crop in South-East Asia, due to an increasing global demand. Current area of rubber plantations is equivalent to 57% of oil palm area globally, with more than 2 million hectares established during last decade^29^. Often, tropical forest is first logged to extract commercially valuable species, and subsequently converted to these non-timber plantations.

### Agroforestry

Agroforestry maintains a structural diversity that imitates the native forest better than conventional pastures, row crops, and monoculture plantations^30^. In agroforestry systems, perennial tree crops such as coconut, rubber, coffee or cacao replace the original forest understorey but some canopy trees are left for shade. Timber from agroforestry systems is rarely sold on international markets, therefore we do not include this management type in our case studies, but include it in the meta-analysis to contextualize other management types. There are many different ways in which agroforestry can be implemented (such as agrosilvopastoral systems, row planting, or companion cropping), and this may also have different effects on biodiversity^31^. Due to limited data availability, we group all agroforestry types together.

### Slash & burn

Also referred to as swidden or shifting agriculture, slash-and-burn describes an agricultural system in which forest is periodically cleared and burnt to create fields for crops such as rice, cassava, maize, and papaya. After several years of production, the fields are left fallow, and forest typically regenerates to some extent. Whereas slash-and-burn management rarely contributes timber to international markets, it does provide wood for local subsistence^32^.

## Objective & Scope

To understand the variable impacts of forestry management types on local species richness of different taxa, we conduct a global categorical meta-analysis, using log response ratio (logarithm of the ratio of species richness in managed to unmanaged forest) as a measure of effect size^33^. Existing meta-analyses on the subject are either restricted to a region (e.g. Europe^11^, the tropics^34^), taxon (e.g. plants^35^), management type (e.g. selective logging^23^), or consider forestry as one generic land use type without accounting for differences in management types^36^. We first derive management, taxa-, and continent-specific effect sizes.

To evaluate trade-offs between economic profitability and biodiversity conservation, we consider 10 hypothetical wood-producing Forest Management Units (FMUs) from around the globe, each 5000 ha in size. We calculate the economic profitability of timber harvesting in terms of the net present value of each FMU, over an operation period of 75 years. Our hypothetical FMUs are based on published studies that report the data on management type, harvesting intensity, rotation length, timber species and production costs. The NPV for each FMU was calculated by discounting (10% interest rate) the revenues as well as the production costs associated with future rotation cycles. Net profit over the operation period of 75 years was calculated by multiplying NPV with the area of each FMU (i.e. 5000 ha) in million$.

Next we use the *local* species richness loss estimates derived from our meta-analysis to calculate the *regional* species loss through countryside species-area relationship^37^. By comparing the regional species loss with net profit in the hypothetical FMUs, we illustrate biodiversity-economic trade-offs connected with different forestry management types.

## Results

We compiled 1008 species richness comparisons of managed and unmanaged forests from a total of 287 studies, and used these in an unweighted^38^ meta-analysis (see Supplementary spreadsheet online for raw data). The overall effect size showed that forest management leads to a 29% reduction in species richness (95% CI = 26 to 32%; Supplementary Table S1).

### Management type

**Figure 1:**
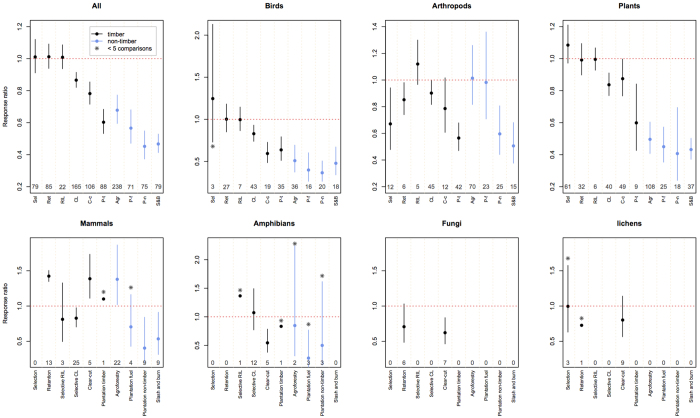
Changes in species richness of different taxonomic groups in response to ten most common forest management regimes. Points represent mean response ratios and lines 95% confidence intervals. The top left panel shows responses for all taxa combined. The management regimes under which timber production is the main goal are showed in black, and non-timber regimes in blue. Horizontal red line signifies no change in species richness. An asterisk designates response ratios based on five or fewer comparisons. Numbers close to the x-axis are the numbers of comparisons. See also Supplementary Table S2 online.

We grouped the compiled dataset into 10 management categories and calculated the effect size for each group. Three out of six timber producing management regimes - Selection and Retention systems and Reduced Impact Logging - did not alter species richness (Fig. 1). Out of the other three timber producing management types, timber plantations had the highest impact (on average 40% reduction in local species richness compared to natural forests), followed by clear-cutting (22% reduction), and conventional selective logging (13% reduction, Fig. 1).

Out of the non-timber producing management regimes that we considered, agroforestry was least detrimental, retaining on average 68% of species richness found in nearby natural forests. Fuel wood plantations had a very similar impact on species richness as timber plantations (i.e. ~40% reduction). Non-timber tree plantations, referring mostly to oil palm and rubber, and slash & burn reduced the species richness by ~54% which is highest impact among all the 10 management systems considered (Fig. 1; Supplementary Table S1).

### Management type ×Taxa

We calculated the effect size for each combination of management type and taxonomic group (Fig. 1). For most management types, the mean effect sizes differed across taxa, which means that taxa respond differently to a particular management type (*P* < 0.001, ANOVA test, Supplementary Table S2). The non-timber producing forest management types reduced bird richness more seriously than when richness of all taxa was considered together (Fig. 1). In contrast to the case when all taxa were considered together, clear-cuts (<10 years old) were worse in terms of bird species richness than timber plantations. Only two timber-producing management categories, retention and timber plantations, caused a decrease in species numbers for arthropods (Fig. 1). Both clear-cutting and conventional selective logging decreased arthropod species richness somewhat, but not significantly. Equally, non-timber producing management regimes had less severe impacts on arthropods. Agroforestry and fuel plantations did not decrease arthropod species richness. All timber producing regimes maintained high plant species richness, except timber plantations, which performed similarly to agroforests.

There was a paucity of studies on mammals, amphibians, fungi and lichens for most management types. Mammalian richness was decreased by conventional selective logging, slash & burn, and non-timber plantations. Retention systems and agroforests did not lower the mammalian species richness. Amphibian richness was on average not affected by conventional logging and the spread of responses was very wide. Fungal diversity was lower under both retention forestry and clear-cutting, and lichen diversity did not change under any management regime for which data was available.

### Management type ×Region

Next, we grouped management types according to region (continent). Not all forest management types are practiced in all regions, nevertheless, some management regimes showed inter-regional variation. For agroforestry, clear-cut, selective logging, timber plantations, and plantations for fuelwood, the mean effect sizes differed among continents (Supplementary Table S3). Timber plantations in Africa maintained on average higher species richness than those on other continents. Other timber producing management regimes did not show a vast difference in impacts between continents. For example, clear-cutting in North America had a very similar effect on species richness as in Europe. Fuel plantations performed similarly on all continents with tropical forests (Africa, Asia, and South America). Non-timber plantations had lower species richness in Africa, Asia, and Central America, when compared with non-timber plantations of South America. Equally, agroforestry in South America caused smaller species losses than in Africa, Asia, and Central America.

### Management type ×Region ×Taxa

We further sub-divided the above management type ×region groups according to the taxon. The majority of effect sizes for this three-way combination could not be calculated or were non-significant owing to missing or low number of comparisons available (Supplementary Table S4). However, for most combinations of management type and continent, we found differences among the mean effect size of taxa (*P* < 0.001, ANOVA test), implying that a taxonomic group of a given continent responds differently to the different management types.

### Biodiversity-economic trade-offs

**Figure 2:**
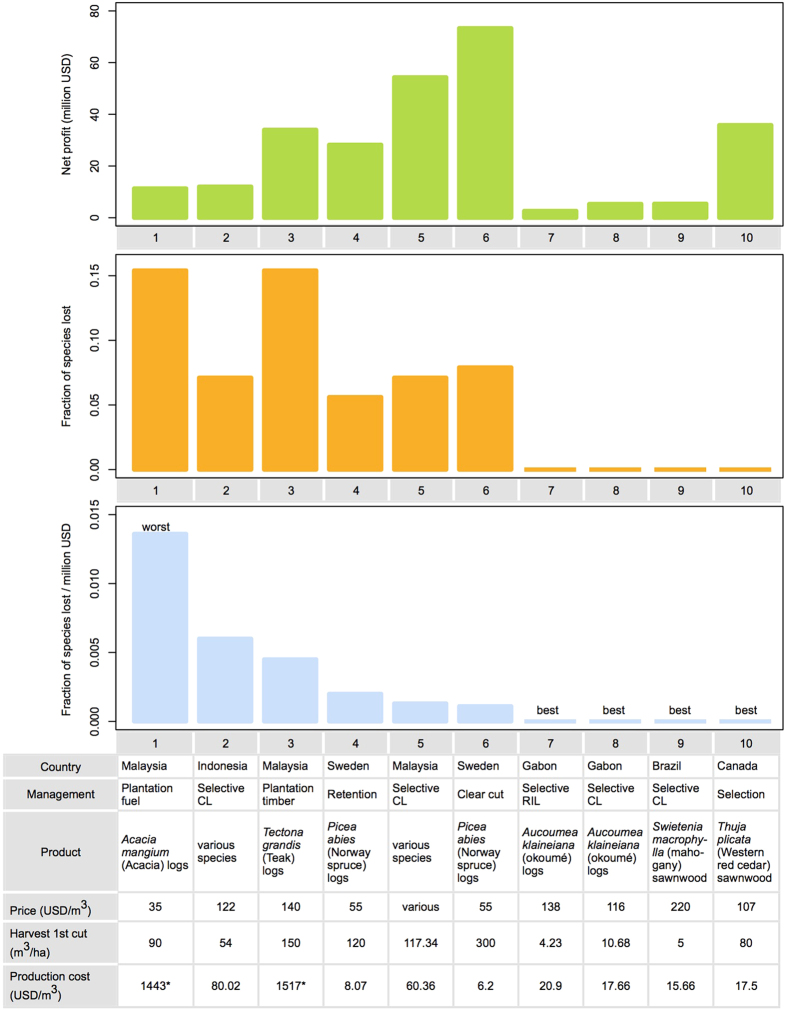
Biodiversity-economic trade offs for ten hypothetical forest management units (FMU), described in the table at the bottom. The top panel shows Net Present Value per hectare, assuming 75 years of management, and 10% discount rate. The central panel shows fraction of regional species lost when half of the original 10,000 ha of natural forest is subjected to forest management. The bottom panel shows the fraction of species lost per net profit, combining information from the two upper panels. See also Supplementary Table S5 online. *signifies FMUs for which production costs are specified in USD/ha, instead of USD/m^3^.

The 10 hypothetical 5000 ha FMUs differed substantially in the fraction of regional species lost, profit generated, and the ratio thereof (Fig. 2). In terms of net present value (NPV), the clear-cutting of *Picea abies* in Sweden with high timber yields (300 m^3^/ha) came first, generating 14659 $/ha, followed by selective logging in Malaysia, which generated 10855 $/ha (Supplementary Table S5). The FMU in Gabon extracting *Aucoumea klaineiana* (oukoumé) with reduced impact logging techniques had the lowest NPV (521$/ha), mostly due to low harvest per ha (4.23 m^3^/ha in first cut).

When the FMUs were ranked according to fraction of species lost per unit profit, *Acacia mangium* fuel wood plantations in Malaysia performed the worst, causing 15% species loss, whilst generating 11.3 million$ profit over 75 years of operation. This is due to the low price of *Acacia* logs (35$/m^3^) as well as relatively high production costs. Selective conventional logging in Indonesia’s forests caused a lower fraction of species lost (7.1%) but still performed the second worst, owing to high production costs (80.02$/m^3^) associated with timber harvesting. The Indonesian FMU performed worse than a similar, selectively logged FMU in Malaysia’s dipterocarp forest, even though both resulted in a similar fraction of species lost. The difference was due to higher yields and lower production costs in the Malaysian FMU, (Supplementary Tables S5 and S6).

It is interesting to see that although the *Tectona grandis* (teak) timber plantations in Malaysia resulted in the highest fraction of species lost (15.4%) among all FMUs considered, it still performed better than the Malaysian fuel plantation and Indonesian selective logging FMU, because of high yields (150 m^3^/ha) and high timber price (140$/m^3^). Similarly, although the clear cutting of *Picea abies* in Sweden caused a higher fraction of species loss (7.6%) than a similar FMU in Sweden, operating with a much more biodiversity benign shelterwood retention (5.5% species loss), it generated the highest net income of all FMUs (73.3 million$), and thus resulted in a lower species loss per million$ than the retention system.

No species loss occurred in the case studies corresponding to Reduced Impact Logging of *Acoumea klaineiana* in Gabon and selection system cutting of *Thuja plicata* in Canada (Fig. 1). When adjusted for logging intensity, conventional selective logging of *Acoumea klaineiana* in Gabon and *Swietenia macrophylla* in Brazil also caused almost no species loss (Supplementary Table S6).

## Discussion

All types of management change some properties of the forest, such as tree age structure, microclimate, or soil conditions. It is unrealistic to expect any type of forestry to have no impact on forest biodiversity. We demonstrated that these impacts vary substantially between forest management types. Through case studies, we illustrated that producing the same economic worth of wood in different ways may result in substantially different biodiversity loss.

We found that in terms of relative species loss at plot level, ways to produce timber can be ranked from best to worse as follows: selection and retention systems in temperate and boreal regions, reduced impact selective logging (RIL) followed by conventional selective logging in tropical regions, clear-cutting in temperate regions, and timber plantations (Fig. 1). Management regimes not focusing on timber production are in general more harmful to species richness than timber producing regimes. A notable exception is agroforestry, which is associated with lower species loss than timber plantations.

Management regimes that mimic small-scale disturbance such as selection harvest, retention, and RIL, result in increased environmental heterogeneity compared to clear cutting, timber plantations or high intensity conventional logging^18^,^39^. Selection and retention systems appear to be similarly good at maintaining high species richness in managed forests. The retention trees, areas, and unharvested portions of selections systems provide late successional structures that are more likely to maintain high diversity. Retained trees also enhance habitat connectivity over the landscape and supply refuges for species after harvest^40^. However, the effect of each of these management types can vary with the proportion of trees retained, and the time since harvest^15^.

Conventional selective logging reduces species richness more than RIL (Fig. 1). However, in the case of trees, this may be due to the lower logging intensities typically associated with RIL^41^. In the case of fauna, which is also strongly influenced by logging intensity, RIL has nevertheless been found to have a smaller impact on biodiversity even after taking logging intensity into account^42^,^43^.

Interestingly, we found species richness differences between timber, fuel wood, and non-timber plantations (mainly oil-palm and rubber). Whereas plant species richness was similar, fuel wood plantations were poorer in bird species than timber plantations. Birds may prefer to nest in timber plantations, as fuel wood plantations are typically harvested more frequently, perhaps not allowing the trees to grow to sufficient sizes suitable for nesting.

Slash-and-burn agriculture in the tropics was found to have higher impacts on species richness than clear-cutting in temperate regions, even though both systems are based on removing all trees from small forest patches. This could be due to the additional effect of burning or the temporary presence of agricultural crops, which could affect the soil and slow down regeneration^44^. Additionally, tropical forests have a higher proportion of forest specialist species, that may be more sensitive to the removal of forest patches, habitat change, and edge effects^45^. However, to truly understand the effect of slash-and-burn agriculture, biodiversity would have to be studied at a broader scale. Certain swidden agricultural systems can provide landscape level biodiversity benefits and be important components of a ‘conservation-agriculture matrix’^32^.

Finally, we found agroforestry to be positioned between timber and non-timber producing regimes in terms of species richness (Fig. 1). Biodiversity within agroforestry systems is enhanced when there is high diversity of native trees in the canopy, low chemical inputs are used, and the understory clearing is not intense^46^.

The variability in the response of different taxonomic groups to forest management is likely both due to variations in biophysical and life history traits, and due to local context of forestry interventions^43^,^47^. For example, primates with slow reproduction and low ecological flexibility show a more marked decline after forest disturbance^47^. Bird populations are negatively affected by reduction in tree cavities for nesting, absence of leaf-litter microfauna, and foraging sites in the logged forest^48^. Equally, the amount of dead wood, large logs and snags of different decay stages post-logging determine the survival of many taxa^11^. We expect that insectivores do better in fuel wood plantations, due to the high abundance of arthropods (Fig. 1) but not frugivorous who need fleshy fruit trees for survival.

Plants showed mixed response to logging. Many studies reported an increase in species richness following logging, which may be a result of early successional colonizers with a negative effect on the late successional shade tolerant species^35^. Similarly, bryophytes might respond differently to forest management than vascular plants^11^, however, more data is needed to tease out such differences. Mammal diversity appeared to benefit from forest disturbance (Fig. 1). This disparity might be because of high tolerance of certain mammal species to degraded forests^49^, particularly among bats and small forest floor mammals. Indeed, out of a total of 91 comparisons of mammals compiled in this study, 82 were for either small mammals or bats.

When we evaluated the timber producing management regimes in terms of species loss per unit profit from wood harvesting in our selection of case studies, their rankings changed. For example, while the meta-analysis revealed that conventional selective logging is less harmful to species richness than timber plantations, the Indonesian FMU operating with conventional selective logging resulted in a higher fraction of species lost per million$ profit generated than a *Tectona grandis* timber plantation FMU from Malaysia.

Clearly, our case studies cannot be used to generalize outcomes for the selected timber producing management regimes and countries. We made simplifying assumptions on area surrounding the forestry concessions (matrix) and the spatial extent of timber species within FMUs, all of which have high variability both in space and time. Data for timber prices, yield, rotation cycle, and production costs are highly site-specific and thus caution must be taken while interpreting the results^50^,^51^. For selective logging regimes, the net present value would be higher if the profits from subsequent conversion to another land use type, such as oil palm or rubber plantation were also included. We also did not include the potential revenues from non-timber forest products. Further, we assumed that all FMUs are replacing natural forests, while in practice forests previously managed through selective logging might have been switched to, for example, RIL.

As our case-studies are hypothetical, we evaluated the outcomes as a fraction of species lost divided by net income generated. However, from an absolute species loss perspective, a 50% temporary extirpation of species in a Swedish concession, where original species richness is relatively meagre and endemic species almost non-existent, may be considered less serious than even a 5% species loss in a hyper-diverse tropical rain forest. Therefore, a more realistic expression of the biodiversity-economic trade-off would be absolute species lost/million$.

We initially compiled some 350 studies from the literature review but over 150 studies, containing >300 pairwise comparisons of species richness had to be excluded because they did not report summary statistics necessary for a meta-analysis, such as standard deviation or sample size. In the selected dataset we found major data gaps owing to which, effect sizes for several three-way combination of management type, taxa and region could not be calculated (Supplementary Table S3).

Our meta-analysis did not take into account the surrounding habitat types, as these were mostly not always reported by the studies^45^. Despite their likely importance, we could not decouple the primary impacts of forest management on biodiversity (i.e. due to tree removal), from the secondary impacts (e.g. higher rates of bushmeat hunting, livestock grazing, illegal logging and fires through increased accessibility of the forest), as the majority of studies provide no such information. However those studies that explicitly stated the presence of hunting, grazing etc. were not included in the analysis.

We used local species richness (alpha diversity) as a proxy for biodiversity changes due to different types of forest management. An increase in species richness, such as we found in selection and retention systems, and under reduced impact logging, does not necessarily signify enhanced biodiversity value: rare forest interior species could still be lost under these management types, and replaced with generalist, common, or even invasive species^15^,^25^. However, a reduction in species richness, such as in other systems, can be safely interpreted as a negative impact. To complement our results, future global meta-analysis should use indicators that compare compositional changes in the community, e.g., Sørensen’s similarity index^52^ that may be more sensitive to land use impacts than relative species richness.

It would be erroneous to dismiss or prioritize timber production regimes, based solely on their ranking of alpha diversity impacts. First, one has to consider that the impacts on biodiversity at a larger scale and within the context of heterogeneous landscapes may be different than plot-scale effects. For example, our results show that timber and fuel wood plantations reduce local species richness more than any other timber-producing regimes, however, the comparisons we made are all with natural forest. Indeed, plantations, when established in previously degraded and intensively managed areas, may enhance local and regional biodiversity and also contribute to alleviation of pressure on the remaining natural forests. To calculate such impacts at regional scale, we used the countryside species-area relationship (SAR), using data on different species’ affinity to various forest use types that we derived through our meta-analysis^53^.

Second, timber production systems should be judged also in terms of their economic productivity. The combined quantitative assessment of economic and biodiversity consequences of land use change that we carried out can also help inform the debate on whether it is better to convert large extents of natural forests to regimes relatively benign to biodiversity (e.g. RIL) or to convert smaller extents of the forests to intensive production systems, such as timber plantations (i.e. land sharing vs. land sparing^54^). Similar analyses are also needed to help design appropriate payments for biodiversity schemes^55^ and are increasingly relevant for decision makers in countries planning to expand productive areas into natural landscape in near future.

## Materials and Methods

### Data collection

We searched for research articles using the *ISI Web of Science* database (keywords are listed in Supplementary information). From this list, we retained those studies that reported species richness comparisons between a managed forest and an unmanaged forest (i.e. forests that had little or no human disturbance) of an equivalent size or equivalent sampling effort in the same region. For each comparison, we recorded the location, management type, taxon and, when available, standard deviation and sample size. Studies that sampled in forests with a disturbance additional to logging, such as hunting, livestock grazing, mining, burning, etc., were excluded. For clear-cut and slash-and-burn management types, we only retained studies that measured species richness within 10 years of the intervention, as time has a large influence on the recovery of biodiversity^44^.

### Data Treatment and Effect Size Calculations

For each comparison, we tabulated the observed mean species richness (*X*) for both managed (subscript *E*) and unmanaged groups (subscript *C*). We calculated the response ratio (*R* = *X*_*E*_/*X*_*C*_) for each comparison and took its natural logarithm *L* = In *R* *=* In(*X*_*E*_/*X*_*C*_), as a measure of effect size for the meta-analysis owing to its favorable statistical properties^33^.

Traditional meta-analyses weight the individual effect sizes by the inverse of the effect size variance, as studies with higher replication and lower variance are considered more precise, and thus weighted more heavily^33^. However it has been shown that ecological field studies comparing biodiversity in logged and unlogged forests are rife with pseudoreplication (see Ramage *et al*.^56^ and Halme *et al*.^57^). Using pseudoreplicated studies in a meta-analysis typically inflates the effect sizes, because the artificially low variation among replicates is due to interdependent samples of the same species pool^57^. Moreover, weighted meta-analysis requires standard deviation and sample size for each comparison, which is not reported by many field studies. Recent studies have advocated using unweighted meta-analysis to avoid under/over estimation of effect sizes and to include data from more studies^38^. For these reasons, we performed unweighted, fixed effects meta-analysis.

We carried out a categorical meta-analysis to test for the effect of three factors - region (continent), taxon, and management type, on the magnitude of change in species richness between unmanaged and managed forests. We calculated the mean effect size *L*_*j*_ and its standard error (*S*_*j*_) for the entire data set, for each subgroup *j*of the above three variables, for the two-level combination of these three variables (management type × region and management type × taxon), and finally for their three-way combination (management type × region × taxon)^33^.

The confidence interval (*CI*) for *L*_*j*_ is given by:


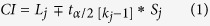


where *t* is the two-tailed value from the Student’s *t*-distribution at the critical level *α* and *k*_*j*_ is the number of pairwise comparisons in the *j*^*th*^ group. The mean effect size for each group (*L*_*j*_) is considered to be statistically significant if its 95% confidence interval does not bracket zero^38^. For the purpose of interpretation we present the results as back-transformed response ratios *R*. We also assessed if statistically significant differences exist between the mean effect sizes for the groups through one-way analysis of variance (ANOVA).

### Species loss and economic trade-offs

We considered 10 hypothetical forest management units (FMUs) operating with different timber producing management regimes in different world regions (sources listed in Supplementary Table S5). For each case, we extracted the following variables from published literature: country, management type, timber species and their yields associated with that management type (m^3^/ha), market price of species (US$/m^3^), production costs (in $/ha or $/m^3^) and rotation cycle (in years).

To estimate the biodiversity cost of timber production in each case, we began with a hypothetical 10,000-ha of existing natural forest in a particular country, out of which 5,000-ha has been allocated to a commercial logging firm as a concession (hereafter referred to as forest management unit, FMU). We then estimated the fraction of regional species lost (*f*_*lost*_) using the countryside species-area relationship (SAR) model^37^,^53^:


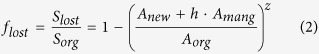


where *S*_*lost*_ is the species loss after forest management intervention; *S*_*lost*_ is the original number of species in the natural forest area; *A*_*org*_ is the original natural forest area with no habitat change; *A*_*mang*_ is the managed forest area (5000 ha); *A*_*new*_ is the remaining natural area (=*A*_*org*_ − *A*_*mang*_ = 5000 ha); the exponent *z* is a constant indicating the rate of change in the number of species per unit area, which we set to be 0.25, as an overall mean for all taxa and continents; and *h* is a measure of affinity of a taxa to the altered habitat (0 < *h* < 1). We derived *h* values for each management regime from our meta-analysis, as 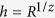 here *R* is the response ratio for each continent-specific management type for all taxa combined (see Pereira *et al*.^37^ for details). For response ratios greater than 1, we assumed that no species loss would occur, and we set *R* as 1. For selective logging case studies, we further adjusted *R* values by taking logging intensity into account. We did this by using an average coefficient, combined for mammals, birds, invertebrates and amphibians, from Burivalova *et al*.^25^ who examined the impact of selective logging intensity on species richness.

Note that countryside SAR predicts the *regional* species loss by considering FMU as a part of heterogeneous landscape surrounded by natural forest. The species loss estimates derived using our meta-analysis are plot-scale, *local* species richness reductions. From equation (2) we can observe that in the extreme case when all natural forest area is converted to a logging concession, the regional and local species loss estimates converge, i.e. when *A*_*mang*_ = *A*_*org*_, *f*_*lost*_ = 1 − *R*.

We then calculated the net present value (*NPV* in US$/ha) from wood extraction in each FMU as follows:


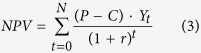


where *P* and *C* are respectively the market price ($/m^3^) and production cost ($/m^3^) of timber species within FMU, *r* is the interest rate (assumed to be 10%) and *t* is the time of cash flow. *N* is the number of rotation cycles over a total of 75 year period and *Y*_*t*_ is the timber yield in a particular rotation cycle (m^3^/ha).

For FMUs operating with selective logging and reduced impact logging techniques we assumed a rotation cycle of 25 years and assumed that timber yields decline by 46% after the first harvest and remain at that level in subsequent rotation cycles^9^. For *Acacia Mangium* fuel plantation and *Tectona grandis* timber plantation FMUs in Malaysia –establishment, maintenance and harvesting costs along with timber yields and prices were taken from existing FAO report^58^ and the rotation cycle was assumed to be 8 years and 15 years respectively^58^. Here we assumed that the timber yields remain the same in each rotation cycle. Data for *Picea abies* FMUs in Sweden operating with clear-cut and retention regimes was imported from Hanell *et al*.^59^, assuming a rotation cycle of 75 years. We selected two *Aucoumea klaineiana* FMUs from Gabon - one operating with selective logging and another with RIL techniques. For these FMUs, production cost data was taken from Medjibe & Putz^50^, while yield data from Medjibe *et al*.^60^. For selectively logged FMUs in Malaysia and Indonesia, the price, yield and production cost data were taken from Fisher *et al*.^55^ and Ruslandi *et al*.^51^ respectively. See Supplementary Table S5 for detailed NPV calculations for each FMU and the data sources used.

NPV was multiplied with total managed forest area (*A*_*mang*_ = 5000 ha) to obtain net profit (in million$) from each FMU over a 75 year period. Finally, we divided the fraction of species lost due to a particular management type in the FMU (equation 2) by the net profit from it. All FMUs were ranked according to their fraction of species lost per unit profit from worst (highest) to best. See Supplementary Table S6 for detailed calculations on tradeoffs.

## Additional Information

**How to cite this article**: Chaudhary, A. *et al*. Impact of Forest Management on Species Richness: Global Meta-Analysis and Economic Trade-Offs. *Sci. Rep*. **6**, 23954; doi: 10.1038/srep23954 (2016).

## Supplementary Material

Supplementary Information
